# Correlates of Meningococcal B Vaccination and Health Behavior Profiles Among MSM in China

**DOI:** 10.3390/vaccines13090983

**Published:** 2025-09-19

**Authors:** Rongyan Li, Qian Zou, Yi Zhou, Ye Zhang, Dan Wu, Xinyuan Zhang, Fengshi Jing, Jie Fan, Xi He, Weiming Tang

**Affiliations:** 1 School of Public Health, Nanjing Medical University, Nanjing 210000, China; 2 University of North Carolina Project-China, Guangzhou 510000, China; 3 Zhuhai Center for Disease Control and Prevention, Zhuhai 519000, China; 4 Faculty of Data Science, City University of Macau, Taipa, Macao SAR, China; 5 Zhuhai Xutong Voluntary Services Center, Zhuhai 519000, China; 6 Dermatology Hospital of Southern Medical University, Guangzhou 510000, China

**Keywords:** meningococcal B vaccine, men who have sex with men (MSM), vaccination coverage, sexual behavior, China

## Abstract

Background: Meningococcal B (MenB) vaccination offers protection against invasive meningococcal disease and moderate cross-protection against gonorrhea. However, little is known about coverage and behavioral correlates among men who have sex with men (MSM) in China. This study assessed self-reported MenB vaccination uptake and its associations with sociodemographic and behavioral factors. Methods: We conducted a nationwide cross-sectional survey among 1022 MSM recruited via community-based organizations and online platforms. Vaccination status and recent sexual behaviors were self-reported. Logistic regression identified correlates of uptake, and latent class analysis (LCA) examined behavioral profiles. Results: Participants had a mean age of 29.6 years; most were unmarried (87.7%) and nearly 90% had a college degree or above. Overall, 21.7% reported receiving MenB vaccination. Uptake was positively associated with condomless anal intercourse (aOR = 1.57, 95% CI: 1.08–2.31), group sex (occasionally: aOR = 1.63, 95% CI: 1.01–2.64; frequently: aOR = 3.86, 95% CI: 1.85–8.04), and female partners in the past six months (aOR = 3.69, 95% CI: 2.25–6.10). MSM with multiple casual male partners were less likely to be vaccinated (aOR = 0.55, 95% CI: 0.32–0.93). LCA identified heterogeneous subgroups; notably, the “multi-partner and proactive” group, with high pre-exposure prophylaxis against HIV infection awareness and frequent STI testing, showed low uptake (13.4%). Conclusions: MenB vaccination coverage among MSM in China remained suboptimal. Uptake differed across behavioral subgroups, underscoring the need for stratified, context-specific strategies to inform future vaccine introduction.

## 1. Introduction

Invasive meningococcal disease (IMD) is a severe, life-threatening infection caused by *Neisseria meningitidis*, with substantial morbidity and mortality worldwide, particularly among vulnerable populations [1]. Men who have sex with men (MSM) are increasingly recognized as a high-risk group, with multiple IMD outbreaks reported in MSM communities across Europe and North America [2,3], and epidemiological data from the United States indicating a fourfold higher risk compared with non-MSM, especially among those living with HIV [4]. Nearly all IMD cases are caused by six major serogroups—A, B, C, W, X, and Y—with serogroup B (MenB) emerging as a predominant cause in adolescents and other high-risk groups [5]. Since 2013, MenB vaccines such as 4CMenB (Bexsero^®^) and MenB-FHbp (Trumenba^®^) have been licensed in Europe, North America, Australia, and Canada, and incorporated into several national immunization programs [6,7]. By contrast, the situation in China is markedly different: no MenB vaccine has yet been approved for marketing or included in the national immunization program, resulting in negligible availability and no routine coverage [8]. Real-world evidence also suggests that 4CMenB may confer cross-protection against MenC and MenW [9,10]. Nevertheless, uptake among high-risk populations, including MSM, remains suboptimal due to limited awareness, structural barriers, and persistent stigma [11,12].

*Neisseria meningitidis* and *Neisseria gonorrhoeae* share approximately 80–90% genome sequence identity [13], providing a biological rationale for the observed cross-protective effects of MenB vaccines against gonorrhea. Several observational studies have reported that MenB vaccination may reduce the risk of gonorrhea by more than 30% [14,15]. Given that MSM are disproportionately affected by both IMD and gonorrhea, and face the additional challenge of increasing antimicrobial resistance for gonorrhea, MenB vaccination may offer dual public health benefits in this population [16,17,18], especially among MSM, in whom the transmission of resistant strains has emerged as a global concern [19,20]. Taken together, these findings highlight the potential of MenB vaccination not only to prevent IMD but also to contribute to mitigating gonorrhea incidence in high-risk populations. In mainland China, while MenA and MenC vaccines are included in the national immunization program, awareness of MenB remains limited due to its absence from routine immunization [8].

Although international attention to MenB vaccination in MSM populations has been increasing, evidence from China remains scarce. To address this gap, we conducted a nationwide, multi-province cross-sectional survey in 2024 to assess self-reported MenB vaccine uptake and its associations with key sociodemographic and sexual behavior characteristics. We also applied latent class analysis to capture heterogeneity in health engagement and compared uptake across behavioral profiles, aiming to provide evidence that may inform future prevention strategies.

## 2. Methods

### 2.1. Study Design and Participants

We conducted a cross-sectional study among MSM in China between November 29, 2024 and February 11, 2025. Data were collected via an anonymous online survey administered using the Sojump platform (Wenjuanxing, https://www.sojump.com/). Participants were recruited through a nationwide convenience sample, primarily via online promotion on WeChat, including official accounts and Moments posts disseminated by several community-based organizations (CBOs) and public health groups across multiple cities.

Eligible participants were biologically male at birth, aged 18 years or older, and reported a history of sexual activity with other men. Quality control procedures included removing duplicate entries based on identical IP addresses or contact information and excluding questionnaires that failed an embedded attention-check question (“Please select number three”). After quality control and removal of duplicates or invalid responses, a final analytic sample of 1022 MSM was included. All participants provided informed consent prior to survey initiation. This study was approved by the ethics committee of Shenzhen University (Approved No.: PN-202400070).

### 2.2. Data Collection

Data were collected using a structured, anonymous online questionnaire via the Sojump platform. The survey included items on sociodemographic characteristics, sexual behaviors, health-related behaviors, and MenB vaccination status.

Sociodemographic variables included age, education level (high school or below, college, graduate or above), monthly income (< $420, $420–1110, >$1110), marital status (single, engaged or married, divorced/separated/widowed), sexual orientation (gay, others), gender status (male, non-male, where ‘non-male’ referred to transgender women or non-binary individuals who were biologically male at birth), and sexual orientation disclosure (not disclosed to anyone, disclosed only to healthcare providers, disclosed only to non-provider individuals). Sexual behavior variables included the number of regular male sexual partners and casual male sexual partners in the past six months (categorized as none, one, or more than one), occurrence of condomless sex in the past six months (yes/no), group sex participation (never, occasionally, frequently), and whether the participant had sex with a female partner in the past six months (yes/no).

We also collected health-related variables, including PrEP awareness (“Before filling out this survey, had you heard of Pre-Exposure Prophylaxis for HIV [abbreviated as PrEP] ?”), frequency of STD testing (categorized as “at least once every 3 months” vs. “once every 6 months or less”), willingness to use long-acting injectable PrEP, and history of STD diagnosis in the past 12 months (yes, no, unsure). For analysis, participants who responded “yes” or “unsure” to STD diagnosis were grouped together, consistent with previous studies

Self-reported MenB vaccination status was assessed with a survey item asking whether participants were “vaccinated”, “partially vaccinated”, or “unvaccinated”. For the main analyses, MenB vaccination status was dichotomized as vaccinated (including partially and fully vaccinated) vs. unvaccinated.

All questionnaire items were self-reported and adapted from validated instruments used in previous MSM studies where possible. The questionnaire was pilot-tested for clarity and comprehensiveness prior to field implementation.

### 2.3. Statistical Analysis

All statistical analyses were performed using R version 4.5.1. Descriptive statistics were used to summarize participant characteristics, MenB vaccination status, and key behavioral variables. Categorical variables were reported as frequencies and percentages, while continuous variables were presented as means and standard deviations or medians and interquartile ranges, as appropriate.

Multivariable models focused on sociodemographic and sexual behavior factors to provide a clear assessment of background and risk-related correlates of MenB vaccination, without adjusting for other health-promoting behaviors that might mediate or confound these associations. To identify factors associated with MenB vaccination, univariate logistic regression analyses were first conducted for each candidate variable. Variables with a *p*-value < 0.10 in univariate analysis were subsequently included in the multivariate logistic regression model. Adjusted odds ratios (aORs) and 95% confidence intervals (CIs) were calculated.

Building on previous research among MSM [21], eight dichotomized variables were selected for the latent class analysis (LCA) to capture both behavioral risk exposure and health-related engagement. The behavioral risk exposure indicators included having multiple regular male partners (yes/no), multiple casual male partners (yes/no), condomless sex in the past six months (yes/no), and participation in group sex (yes/no). The health-related engagement indicators included frequent STD testing (at least once every 3 months vs. less frequent), PrEP awareness (yes/no), and willingness to use long-acting injectable PrEP (interested vs. uninterested). Sexual orientation disclosure (yes/no) was included as an additional indicator reflecting openness about sexual identity and potential access to social support. All variables were dichotomized prior to analysis to facilitate model convergence, interpretability, and comparability.

Models with two to five classes were compared using the Bayesian Information Criterion (BIC), classification entropy (CE), and class interpretability. When fit indices were similar, the final number of classes was selected based on both statistical criteria and substantive interpretability. Each participant was assigned to the class with the highest posterior probability. Subsequently, chi-square tests were performed to compare MenB vaccination status and self-reported STD diagnosis in the past 12 months across latent classes.

## 3. Results

A total of 1022 valid and non-duplicate questionnaires were included in the final analysis. Participants were recruited from 28 provinces across China, with the largest proportions from Guangdong (*n* = 348, 34.1%), Shandong (*n* = 112, 11.0%), and Hubei (*n* = 98, 9.6%). In addition, 12 participants (1.2%) were recruited from regions outside mainland China (e.g., Hong Kong, Macau, Taiwan, or abroad). The full distribution of participants by province is provided in Table S1.

### 3.1. Participant Characteristics

Table 1 summarizes the baseline characteristics of the participants. The mean age was 29.6 years (SD = 7.61). Most were unmarried (87.7%) and held a university degree or higher (87.8%). Over one-third reported a monthly income exceeding $1110 USD. The majority identified as gay (75.0%), and almost all self-identified as male (95.5%).

Regarding sexual behaviors in the past six months, 26.6% reported having more than one regular partner, 30.9% had more than one casual partner, 57.0% engaged in condomless sex, 22.8% participated in group sex, and 13.0% had female sexual partners.

Overall, 222 participants (21.7%) had received at least one dose of the MenB vaccine.

### 3.2. Factors Associated with MenB Vaccination

Increasing age was associated with reduced odds of MenB vaccination (aOR = 0.95 per year increase, 95% CI: 0.92–0.98), indicating that younger participants were more likely to be vaccinated. Reporting high-risk sexual behaviors in the past six months—such as condomless sex (aOR = 1.57, 95% CI: 1.08–2.31), engaged group sex in the past three months (occasionally: aOR = 1.63; frequently: aOR = 3.86), and sex with female partners in the past six months (aOR = 3.69, 95% CI: 2.25–6.10)—was associated with higher vaccine uptake, while having more than one casual male partner was negatively associated with vaccination (aOR = 0.55, 95% CI: 0.32–0.93). Detailed results are presented in Table 2.

### 3.3. Latent Class Analysis of Behavioral Patterns

Eight binary behavioral indicators were included in the LCA, and pairwise Spearman correlation coefficients among them were all below 0.5, indicating no substantial collinearity or redundancy. Following this assessment, latent class models with two to five classes were fitted and compared using the Bayesian Information Criterion (BIC) and classification entropy (CE). As shown in Table S2, the four-class model had the lowest BIC (8833.4) and highest CE (0.997), while the three-class model also demonstrated a low BIC (8835.6) and high CE (0.996).

**Figure 1:**
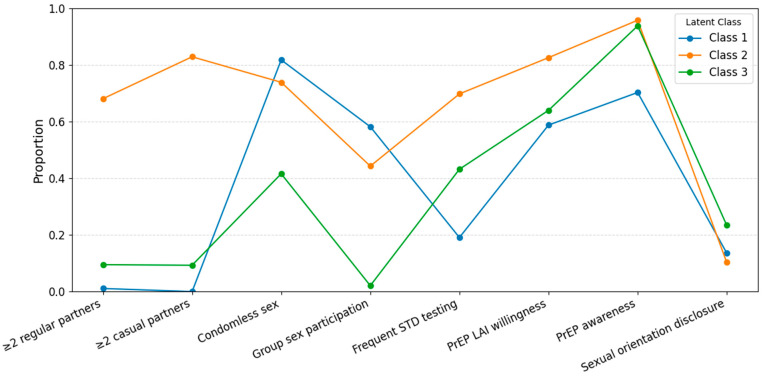
Profiles of health risk and health-promoting behaviors by latent class among MSM (*n* = 1022).

Considering both model fit statistics and substantive interpretability, the three-class solution was selected as the final model for subsequent analyses. Class 1 (“Low-partner, high-risk”, 10.5%) had few partners but relatively high rates of condomless sex and group sex. Class 2 (“Multi-partner, proactive”, 30.1%) had the highest number of regular and casual partners, frequent STD testing, high PrEP awareness and willingness, and engaged in both risk and preventive behaviors. Class 3 (“Low-risk, conservative”, 59.5%) reported the lowest prevalence of high-risk sexual behaviors, the highest rate of sexual orientation disclosure, and moderate preventive behaviors (Figure 1). The detailed behavioral profiles for each class are shown in Figure 1 and Table S3.

### 3.4. Association Between Latent Classes and MenB Vaccination Status

In the “Low-partner, high-risk” class, MenB vaccination coverage was highest at 61.7%, and the proportion with a recent STD diagnosis (or unsure) was also highest at 38.3%. By contrast, the “Multi-partner, proactive” and “Low-risk, conservative” classes showed substantially lower vaccination rates (13.4% and 18.9%) and lower rates of recent STD diagnosis (16.0% and 8.9%, respectively). Both MenB vaccination status and recent STD diagnosis differed significantly across latent classes (chi-square test, *p* < 0.001 for both comparisons) (Table 3). These findings suggest that behavioral subgroup differences may inform the design of more targeted and effective vaccination and sexual health interventions for MSM.

## 4. Discussion

In this nationwide cross-sectional study of MSM in China, we assessed self-reported meningococcal B vaccination coverage and its behavioral correlates. This study extended the limited literature on MenB vaccination among MSM, complementing prior qualitative and meta-analytic findings on suboptimal uptake and its behavioral determinants [11,22]. Among 1022 participants from multiple provinces, 21.7% (222/1022) self-reported having received MenB vaccination. Our findings revealed that self-reported vaccine uptake was associated with specific sexual behaviors, including condomless sex and group sex, and varied across behavioral profiles identified through latent class analysis. These results provide a basis for planning more tailored vaccination strategies.

Our findings indicated a moderate self-reported MenB vaccine uptake, which is broadly consistent with international trends but likely reflects some degree of overestimation. For instance, U.S. data from 2020 showed that only 28.4% of 17-year-olds had received at least one MenB vaccine dose, and series completion rates among insured adolescents remained below 60% [23]. Similarly, MenB coverage among medically vulnerable populations has been reported at 12.1% in Australia and 8.0% in Norway [24,25]. However, such data remain scarce among MSM, particularly in low- and middle-income settings. Since no MenB vaccine is currently licensed in mainland China, the reported uptake should be interpreted cautiously, as it may not fully reflect true coverage. Nevertheless, even if partly inflated, these self-reports may still reflect some willingness to engage with vaccination among MSM. Existing studies have demonstrated that MenB vaccines provide moderate cross-protection against gonorrhea, which is especially relevant for MSM populations facing both a high burden of infection and increasing resistance [14,15,26]. Several high-income countries, including the United States, the United Kingdom, and Australia, have already implemented targeted MenB vaccination strategies for high-risk groups in schools, universities, or outbreak settings [18,27,28,29], offering insights that could inform future considerations in China. In addition, given the marked regional differences in IMD incidence and circulating serogroups reported in China [30], future MenB-related research and vaccination planning would benefit from closer integration of local epidemiological data to better inform tailored strategies.

Within our study population, younger MSM were more likely to report MenB vaccination. This pattern may reflect generational differences in health awareness, willingness to seek preventive services, and greater opportunities to access vaccines through overseas travel or private healthcare settings. In addition, MSM who reported condomless sex or group sex in the past six months had significantly higher self-reported MenB vaccination rates than those without such behaviors. Although the behavioral window was limited, these patterns likely reflect broader risk exposure and behavioral tendencies [31,32]. One plausible explanation is that individuals engaging in higher-risk sexual behaviors may have elevated risk perception and thus be more proactive in adopting preventive measures, including vaccination against *Neisseria meningitidis* and other sexually transmitted infections such as gonorrhea [33,34]. Similarly, MSM who reported sexual activity with female partners also exhibited higher uptake. This group, commonly referred to as MSMW (men who have sex with men and women), tends to be embedded in more complex and higher-risk sexual networks than MSM-only, and may feel a stronger sense of health responsibility toward both male and female partners [35]. Their increased service contact, including through premarital or reproductive health checkups, may also facilitate vaccination. However, prior research in China suggests that MSMW may still underutilize HIV testing and PrEP services [36], highlighting a mismatch between access potential and actual engagement. Importantly, condomless sex, group sex, and bisexual partnerships are also the key driving forces of gonorrhea transmission, underscoring the public health value of planning for MenB vaccination among those most in need [16]. It may be beneficial to integrate MenB vaccination into broader sexual health services that consider both same-sex and opposite-sex sexual partnerships, in order to reach individuals across diverse behavioral profiles, including MSMW.

In contrast, MSM reporting multiple casual male partners showed lower self-reported vaccination rates despite elevated risk. Similar counterintuitive patterns have been observed in HPV and hepatitis B vaccination, where greater sexual risk did not translate into higher vaccine uptake [22,37]. Such patterns suggest that increased risk does not consistently translate into preventive action, and that additional barriers may be at play. In particular, MSM with multiple casual partners may face distinct psychosocial and structural obstacles, including concerns about stigma, privacy, or disclosure, which may hinder vaccine-seeking behavior [38,39]. To prepare for future vaccine introduction in mainland China, it will be important to address these barriers not only by enhancing individual motivation but also by creating supportive service environments, such as leveraging digital platforms to deliver confidential vaccine information.

Latent class analysis further illustrated the behavioral diversity in MenB vaccine uptake among MSM. More than half of participants were classified as “low-risk and conservative,” consistent with prior findings that a majority of MSM still belong to more conservative behavioral profiles in both China and international settings [21,40]. In contrast, individuals in the “multi-partner and proactive” group—characterized by high PrEP awareness and frequent STI testing—showed unexpectedly low MenB vaccination rate (13.4%), despite their engagement with other preventive services. These contrasting patterns suggest that future strategies should be subgroup-specific: while conservative groups may benefit from interventions that raise awareness of MenB risks and benefits, proactive groups may require approaches that optimize the timing and framing of vaccine information. For instance, prior studies have shown that introducing vaccine-related information during high-stress situations, like rapid HIV testing, may reduce receptivity due to increased anxiety [41]. Delivering such information during follow-up visits or through digital platforms may better support acceptance by reducing stress and enhancing engagement. Overall, these insights indicate that promoting MenB vaccination requires not only service bundling but also tailored strategies that account for behavioral heterogeneity within MSM populations. However, because vaccination status was self-reported, the relatively low uptake in the “multi-partner and proactive” group could also reflect more meticulous or honest reporting among participants who are already engaged with other health services. This exploratory observation highlights the need for future studies with objective vaccination records to verify whether it reflects true behavioral differences.

This study is subject to several limitations. First, there is a potential for selection bias, as participants were recruited through a nationwide convenience sample from multiple provinces via online platforms and CBO networks, and the sample distribution may not accurately represent MSM populations across different regions in China. Additionally, CBO-based recruitment may have preferentially attracted MSM who are more health-conscious. Second, all data in this study were self-reported, including sensitive information on sexual behaviors and vaccination history, which introduces potential recall and social desirability bias. The survey was anonymous and self-administered online to minimize discomfort and encourage honest reporting. As MenB vaccines are not yet approved in mainland China, reported uptake likely reflects access through cross-border travel or private healthcare channels, a pattern also seen with HPV vaccination. While these routes explain some coverage, overreporting or misclassification (e.g., confusion with MenACWY) remains possible. Our questionnaire briefly mentioned MenB’s partial protection against gonorrhea, which may have unintentionally increased the perceived desirability of vaccination and contributed to overreporting. Third, the cross-sectional design precludes causal inference, and the exploratory use of regression and latent class analysis should be interpreted cautiously. Despite these limitations, this study provides valuable quantitative evidence on MenB vaccination among MSM in China and offers practical implications for future MenB vaccination campaigns in this key population.

## 5. Conclusions

This study examined self-reported MenB vaccination among MSM in China and suggests a moderate reported uptake with considerable potential for improvement once vaccines become available. Uptake differed across behavioral subgroups: men engaging in condomless sex or group sex were more likely to report vaccination, whereas those with multiple casual partners showed lower reported coverage. While based on self-reported vaccination status from a nationwide convenience sample, these findings provide preliminary insights into behavioral patterns that could inform future, regionally tailored vaccination strategies. Efforts to reduce stigma, enhance accessibility, and integrate MenB vaccination into broader sexual health services remain important priorities.

## Figures and Tables

**Table 1:** Characteristics of the participants in China, 2024 to 2025 (*n* = 1022).

Characteristics	Total (*n* = 1022) %	Vaccinated (*n* = 222) %	Unvaccinated (*n* = 800) %
**Age (mean, SD)**	29.58 (7.61)	26.91 (7.16)	30.32 (7.57)
**Education**			
High School or below	125 (12.2)	29 (13.1)	96 (12.0)
College	739 (72.3)	169 (76.1)	570 (71.2)
Graduate or above	158 (15.5)	24 (10.8)	134 (16.8)
**Monthly income** (USD, $)			
Less than $420	136 (13.3)	33 (14.9)	103 (12.9)
$420–1110	517 (50.6)	119 (53.6)	398 (49.8)
More than $1110	369 (36.1)	70 (31.5)	299 (37.4)
**Marital** **s** **tatus**			
Single	896 (87.7)	188 (84.7)	708 (88.5)
Engaged or married	78 (7.6)	25 (11.3)	53 (6.6)
Divorced/Separated/Widowed	48 (4.7)	9 (4.1)	39 (4.9)
**Sexual** **o** **rientation**			
Gay	767 (75.0)	138 (62.2)	629 (78.6)
Others	255 (25.0)	84 (37.8)	171 (21.4)
**Gender status**			
Male	976 (95.5)	196 (88.3)	780 (97.5)
Non-Male	46 (4.5)	26 (11.7)	20 (2.5)
**Sexual orientation disclosure**			
Not disclosed to anyone	184 (18.0)	33 (14.9)	151 (18.9)
Disclosed only to healthcare providers	598 (58.5)	146 (65.8)	452 (56.5)
Disclosed only to non-healthcare individuals	240 (23.5)	43 (19.4)	197 (24.6)
**Number of regular male sexual partners (past 6 months)**			
No regular partner	163 (15.9)	28 (12.6)	135 (16.9)
One regular partner	587 (57.4)	160 (72.1)	427 (53.4)
More than one regular partner	272 (26.6)	34 (15.3)	238 (29.8)
**Number of casual male sexual partners (past 6 months)**			
No casual partner	308 (30.1)	62 (27.9)	246 (30.8)
One casual partner	398 (38.9)	123 (55.4)	275 (34.4)
More than one casual partner	316 (30.9)	37 (16.7)	279 (34.9)
**Condomless sex in the past 6 months**			
No	439 (43.0)	69 (31.1)	370 (46.2)
Yes	583 (57.0)	153 (68.9)	430 (53.8)
**Group sex participation (past 6 months)**			
Never participated	789 (77.2)	142 (64.0)	647 (80.9)
Occasionally participated	189 (18.5)	49 (22.1)	140 (17.5)
Frequently participated	44 (4.3)	31 (14.0)	13 (1.6)
**Sex with a female**			
No	889 (87.0)	146 (65.8)	743 (92.9)
Yes	133 (13.0)	76 (34.2)	57 (7.1)

**Table 2:** Results of multivariable logistic regression analysis for correlates of MenB vaccination among MSM in China, 2024–2025 (*n* = 1022).

Variable	Participants (*n* = 1022)	
	OR (95% CI)	aOR (95% CI)
**Age (mean, SD)**	0.93 (0.9–0.95) ***	0.95 (0.92–0.98) ***
**Education**		
High School or below	Ref.	Ref.
College	0.98 (0.63–1.56)	0.89 (0.53–1.52)
Graduate or above	0.59 (0.32–1.08)	0.66 (0.33–1.3)
**Monthly income** (USD, $)		
Less than $420	Ref.	
$420–1110	0.93 (0.6–1.47)	
More than $1110	0.73 (0.46–1.18)	
**Marital status**		
Single	Ref.	Ref.
Engaged or married	1.78 (1.06–2.91) *	1.56 (0.77–3.11)
Divorced/Separated/Widowed	0.87 (0.39–1.75)	1.69 (0.66–4.03)
**Sexual orientation**		
Gay	Ref.	Ref.
Others	2.24 (1.62–3.08) ***	1.29 (0.84–1.94)
**Gender status**		
Male	Ref.	Ref.
Non-Male	5.17 (2.84–9.56) ***	1.77 (0.8–3.88)
**Sexual orientation disclosure**		
Not disclosed to anyone	Ref.	Ref.
Disclosed only to healthcare providers	1.48 (0.98–2.28)	1.62 (1–2.66)
Others (non-provider only)	1 (0.61–1.66)	0.8 (0.44–1.45)
**Number of regular male sexual partners (past 6 months)**		
No regular partner	Ref.	Ref.
One regular partner	1.81 (1.17–2.87) **	1.29 (0.76–2.25)
More than one regular partner	0.69 (0.4–1.19)	0.7 (0.37–1.35)
**Number of casual male sexual partners (past 6 months)**		
No casual partner	Ref.	Ref.
One casual partner	1.77 (1.25–2.53) **	1.13 (0.75–1.69)
More than one casual partner	0.53 (0.34–0.81) **	0.55 (0.32–0.93) *
**Condomless sex in past 6 months**		
No	Ref.	Ref.
Yes	1.91 (1.4–2.63) ***	1.57 (1.08–2.31) *
**Group sex participation (past 6 months)**		
Never participated	Ref.	Ref.
Occasionally participated	1.59 (1.09–2.3) *	1.63 (1.04–2.55) *
Frequently participated	10.87 (5.67–21.99) ***	3.86 (1.62–9.46) **
**Sex with female**		
No	Ref.	Ref.
Yes	6.79 (4.62–10.02) ***	3.69 (2.25–6.1) ***

Note: *** *p*  <  0.001, ** *p*  <  0.01, * *p*  <  0.05.

**Table 3:** Distribution of MenB vaccination status and recent STD diagnosis across latent classes among MSM in China, 2024–2025 (*n* = 1022).

Latent Class (*n*)	Unvaccinated *n* (%)	Vaccinated *n* (%)	χ^2^/*p*-Value	No Recent STD *n* (%)	Recent STD/Unsure *n* (%)	χ^2^/*p*-Value
Low-partner, high-risk (*n* = 107)	41 (38.3)	66 (61.7)		66 (61.7)	41 (38.3)	
Multi-partner, proactive (*n* = 307)	266 (86.6)	41 (13.4)		258 (84.0)	49 (16.0)	
Low-risk, conservative (*n* = 608)	493 (81.1)	115 (18.9)		554 (91.1)	54 (8.9)	
Total	800 (78.3)	222 (21.7)		878 (85.9)	144 (14.1)	
χ^2^/*p*-value			115.94/<0.001			66.4/<0.001

Note: χ^2^ refers to chi-square test statistic.

## Data Availability

The datasets generated and/or analyzed during this study are not publicly available due to ethical and privacy considerations, as the data contain sensitive information about participants. De-identified data may be made available upon reasonable request to the corresponding author, Weiming Tang (weiming_tang@med.unc.edu), and subject to institutional and ethical approval.

## Supplementary Materials

The following supporting information can be downloaded at: https://www.mdpi.com/article/10.3390/vaccines13090983/s1. Table S1: Proportion of the Province of the participants; Table S2: Model fit statistics for latent class analysis (LCA) models with different numbers of classes among MSM participants; Table S3: Conditional probabilities of each behavioral indicator by latent class (*n* = 1022).

## Abbreviations

The following abbreviations are used in this manuscript:

aOR	adjusted Odds Ratio
CBO	Community-Based Organization
CI	Confidence Interval
HIV	Human Immunodeficiency Virus
IMD	Invasive Meningococcal Disease
LCA	Latent Class Analysis
MenB	meningococcal serogroup B
MSM	men who have sex with men
MSMW	men who have sex with men and women
PrEP	Pre-Exposure Prophylaxis
STD	sexually transmitted disease
STI	sexually transmitted infection

## References

[1] Martinón-Torres F. (2016). Deciphering the Burden of Meningococcal Disease: Conventional and Under-recognized Elements. J. Adolesc. Health.

[2] Kamiya H., MacNeil J., Blain A., Patel M., Martin S., Weiss D., Ngai S., Ezeoke I., Mascola L., Civen R. (2015). Meningococcal disease among men who have sex with men—United States, January 2012–June 2015. MMWR Morb. Mortal. Wkly. Rep..

[3] Hellenbrand W., Claus H., Schink S., Marcus U., Wichmann O., Vogel U. (2016). Risk of Invasive Meningococcal Disease in Men Who Have Sex with Men: Lessons Learned from an Outbreak in Germany, 2012–2013. PLoS ONE.

[4] Folaranmi T.A., Kretz C.B., Kamiya H., MacNeil J.R., Whaley M.J., Blain A., Antwi M., Dorsinville M., Pacilli M., Smith S. (2017). Increased Risk for Meningococcal Disease Among Men Who Have Sex With Men in the United States, 2012–2015. Clin. Infect. Dis..

[5] Shen S., Findlow J., Peyrani P. (2024). Global Epidemiology of Meningococcal Disease-Causing Serogroups Before and After the COVID-19 Pandemic: A Narrative Review. Infect. Dis. Ther..

[6] Banzhoff A. (2017). Multicomponent meningococcal B vaccination (4CMenB) of adolescents and college students in the United States. Ther. Adv. Vaccines.

[7] Perez J.L., Absalon J., Beeslaar J., Balmer P., Jansen K.U., Jones T.R., Harris S., York L.J., Jiang Q., Radley D. (2018). From research to licensure and beyond: Clinical development of MenB-FHbp, a broadly protective meningococcal B vaccine. Expert. Rev. Vaccines.

[8] Xu J., Chen Y., Yue M., Yu J., Han F., Xu L., Shao Z. (2022). Prevalence of *Neisseria meningitidis* serogroups in invasive meningococcal disease in China, 2010–2020: A systematic review and meta-analysis. Hum. Vaccin. Immunother..

[9] Ladhani S.N., Campbell H., Andrews N., Parikh S.R., White J., Edelstein M., Clark S.A., Lucidarme J., Borrow R., Ramsay M.E. (2021). First Real-world Evidence of Meningococcal Group B Vaccine, 4CMenB, Protection Against Meningococcal Group W Disease: Prospective Enhanced National Surveillance, England. Clin. Infect. Dis..

[10] Castilla J., García Cenoz M., Abad R., Sánchez-Cambronero L., Lorusso N., Izquierdo C., Cañellas Llabrés S., Roig J., Malvar A., González Carril F. (2023). Effectiveness of a Meningococcal Group B Vaccine (4CMenB) in Children. N. Engl. J. Med..

[11] Naidu J., Polonijo A.N. (2023). Barriers and facilitators to HPV and meningococcal vaccination among men who have sex with men: A qualitative study. BMC Public Health.

[12] Wang C., Wang Y., Huang X., Li X., Zhang T., Song M., Wu L., Du J., Lu X., Shao S. (2012). Prevalence and factors associated with hepatitis B immunization and infection among men who have sex with men in Beijing, China. PLoS ONE.

[13] Ruiz García Y., Sohn W.Y., Seib K.L., Taha M.K., Vázquez J.A., de Lemos A.P.S., Vadivelu K., Pizza M., Rappuoli R., Bekkat-Berkani R. (2021). Looking beyond meningococcal B with the 4CMenB vaccine: The Neisseria effect. NPJ Vaccines.

[14] Abara W.E., Bernstein K.T., Lewis F.M.T., Pathela P., Islam A., Eberhart M., Cheng I., Ternier A., Sanderson Slutsker J., Madera R. (2023). Healthy Vaccinee Bias and MenB-FHbp Vaccine Effectiveness Against Gonorrhea. Sex. Transm. Dis..

[15] Abara W.E., Bernstein K.T., Lewis F.M.T., Schillinger J.A., Feemster K., Pathela P., Hariri S., Islam A., Eberhart M., Cheng I. (2022). Effectiveness of a serogroup B outer membrane vesicle meningococcal vaccine against gonorrhoea: A retrospective observational study. Lancet Infect. Dis..

[16] Lyu H., Tang H., Feng Y., Hu S., Wang Y., Zhou L., Huang S., Li J., Zhu H., He X. (2024). Incidence and spontaneous clearance of gonorrhea and chlamydia infections among men who have sex with men: A prospective cohort study in Zhuhai, China. Front. Public Health.

[17] Tao G., Patel C.G., He L., Workowski K. (2024). STI/HIV testing, STIs, and HIV PrEP use among men who have sex with men (MSM) and men who have sex with men and women (MSMW) in United States, 2019–2022. Clin. Infect. Dis..

[18] Abitbol V., Martinón-Torres F., Taha M.K., Nolan T., Muzzi A., Bambini S., Borrow R., Toneatto D., Serino L., Rappuoli R. (2024). 4CMenB journey to the 10-year anniversary and beyond. Hum. Vaccin. Immunother..

[19] Kwong J.C., Chow E.P.F., Stevens K., Stinear T.P., Seemann T., Fairley C.K., Chen M.Y., Howden B.P. (2018). Whole-genome sequencing reveals transmission of gonococcal antibiotic resistance among men who have sex with men: An observational study. Sex. Transm. Infect..

[20] Lewis D.A. (2013). The role of core groups in the emergence and dissemination of antimicrobial-resistant N gonorrhoeae. Sex. Transm. Infect..

[21] Chiou P.Y., Tsao W.W., Lin K.C., Fang Y.Y., Lin K.Y., Li C.L. (2023). Risk and Protective Profile of Men Who Have Sex With Men Using Mobile Voluntary HIV Counseling and Testing: Latent Class Analysis. JMIR Public Health Surveill..

[22] Nadarzynski T., Frost M., Miller D., Wheldon C.W., Wiernik B.M., Zou H., Richardson D., Marlow L.A.V., Smith H., Jones C.J. (2021). Vaccine acceptability, uptake and completion amongst men who have sex with men: A systematic review, meta-analysis and theoretical framework. Vaccine.

[23] Pingali C., Yankey D., Elam-Evans L.D., Markowitz L.E., Williams C.L., Fredua B., McNamara L.A., Stokley S., Singleton J.A. (2021). National, Regional, State, and Selected Local Area Vaccination Coverage Among Adolescents Aged 13–17 Years—United States, 2020. MMWR Morb. Mortal. Wkly. Rep..

[24] de Oliveira Costa J., Gianacas C., Beard F., Gonzalez-Chica D., Chidwick K., Osman R., MacIntyre C.R., Havard A. (2021). Cumulative annual coverage of meningococcal B vaccination in Australian general practice for three at-risk groups, 2014 to 2019. Hum. Vaccin. Immunother..

[25] Orangzeb S., Watle S.V., Caugant D.A. (2023). Adherence to vaccination guidelines of patients with complete splenectomy in Norway, 2008–2020. Vaccine.

[26] Looker K.J., Booton R., Begum N., Beck E., Shen J., Turner K.M.E., Christensen H. (2023). The potential public health impact of adolescent 4CMenB vaccination on Neisseria gonorrhoeae infection in England: A modelling study. BMC Public Health.

[27] Blackwell C.W. (2017). Meningococcal Vaccination in Men Who Have Sex with Men. Public Health Nurs..

[28] MacNeil J.R., Rubin L., Folaranmi T., Ortega-Sanchez I.R., Patel M., Martin S.W. (2015). Use of Serogroup B Meningococcal Vaccines in Adolescents and Young Adults: Recommendations of the Advisory Committee on Immunization Practices, 2015. MMWR Morb. Mortal. Wkly. Rep..

[29] Findlow J., Nuttens C., Kriz P. (2019). Introduction of a second MenB vaccine into Europe—needs and opportunities for public health. Expert. Rev. Vaccines.

[30] Nazhaerbieke H., Fu W., Lan Z., Muheiyati Y., Tian T., Wuqierjiafu C., Xie N. (2025). Epidemiological characteristics of invasive meningococcal disease and carriage prevalence of Neisseria meningitidis in the Xinjiang Uygur Autonomous Region, China, 2004–2023: A retrospective study. PeerJ.

[31] Blumenthal J., Moore D.J., Jain S., Sun X., Ellorin E., Corado K., Hoenigl M., Dube M., Haubrich R., Morris S.R. (2019). Recent HIV Risk Behavior and Partnership Type Predict HIV Pre-Exposure Prophylaxis Adherence in Men Who Have Sex with Men. AIDS Patient Care STDS.

[32] Dombrowski J.C., Harrington R.D., Golden M.R. (2013). Evidence for the long-term stability of HIV transmission-associated sexual behavior after HIV diagnosis. Sex. Transm. Dis..

[33] Jongen V.W., Groot Bruinderink M.L., Boyd A., Koole J.C.D., Teker B., Dukers-Muijrers N.H.T.M., Evers Y.J., Schim van der Loeff M.F., Prins M., de Vries H.J.C. (2024). What determines mpox vaccination uptake? Assessing the effect of intent-to-vaccinate versus other determinants among men who have sex with men. Vaccine.

[34] Djiadeu P., Smith M.D.R., Kushwaha S., Odhiambo A.J., Absalom D., Husbands W., Tharao W., Regan R., Sa T., Zhang N. (2020). Social, Clinical, and Behavioral Determinants of HIV Infection and HIV Testing among Black Men in Toronto, Ontario: A Classification and Regression Tree Analysis. J. Int. Assoc. Provid. AIDS Care.

[35] Russ S., Zhang C., Przybyla S., Liu Y. (2023). Racial Differences of Psychosocial Characteristics, HIV Risk-Taking and HIV Prevention Uptake between Men Who Have Sex with Men Only and Men Who Have Sex with Men and Women: A Community-Based Study in Two US Cities. J. Homosex..

[36] Guo Y., Li X., Song Y., Liu Y. (2012). Bisexual behavior among Chinese young migrant men who have sex with men: Implications for HIV prevention and intervention. AIDS Care.

[37] Mann-Barnes T., Bhimla A., Coronado M., Lin T., Duro-Aina A., Park H., Ma G.X. (2023). Factors that Predict HPV Vaccination Behavior Among Young Men-Who-Have-Sex-with-Men in the Greater Philadelphia Region. J. Racial Ethn. Health Disparities.

[38] Richardson S., Seekaew P., Koblin B., Vazquez T., Nandi V., Tieu H.V. (2017). Barriers and facilitators of HIV vaccine and prevention study participation among Young Black MSM and transwomen in New York City. PLoS ONE.

[39] Cleva M., Gaspari V., Ceccarelli A., Pianese G., Griffa D., Orioni G., Cintori C., Diegoli G., Gori D., Montalti M. (2024). HPV Vaccine Awareness and Uptake Among Sexually Transmitted Infections Clinic Users: A Cross-Sectional Study in Bologna, Italy. Int. J. Environ. Res. Public Health.

[40] Liu Y., Liu X., Wei S., Cheng Z., Xian Y., Zhao Y., Ma J., Chen J., Chen Z., Yang J. (2024). Identifying patterns of sexual behaviors and PrEP uptake characteristics among MSM who were eligible for PrEP: A national cross-section study. J. Virus Erad..

[41] Polonijo A.N., Sein S., Maldonado R., Delos Santos J., Brown B. (2022). Promoting vaccination during rapid HIV testing: Recommendations from men who have sex with men in California. Health Soc. Care Community.

