# Supplementary material for: Correlates of Meningococcal B Vaccination and Health Behavior Profiles Among MSM in China
Source: Vaccines (Basel). 2025 Sep 19;13(9):983. doi: 10.3390/vaccines13090983 (PMC12474423; doi:10.3390/vaccines13090983)
Supplement: Supplementary file 1 [file vaccines-13-00983-s001.zip › vaccines-3880595-supplementary.pdf]

Table S1. Proportion of the Province of the participants

Province	Count	Percent
Guangdong	348	34.1%
Shandong	112	11.0%
Hubei	98	9.6%
Hunan	52	5.1%
Fujian	38	3.7%
Guangxi	37	3.6%
Jiangsu	36	3.5%
Sichuan	34	3.3%
Zhejiang	29	2.8%
Beijing	27	2.6%
Henan	27	2.6%
Shanghai	25	2.4%
Chongqing	24	2.4%
Jiangxi	23	2.3%
Hebei	20	2.0%
Anhui	16	1.6%
Liaoning	12	1.2%
Shanxi	11	1.1%
Foreign	8	0.8%
Shaanxi	8	0.8%
Yunnan	6	0.6%
Xinjiang	6	0.6%
Inner Mongolia	5	0.5%
Hainan	5	0.5%
Guizhou	3	0.3%
Heilongjiang	3	0.3%
Tianjin	2	0.2%
Macau	2	0.2%
Taiwan	1	<0.1%
Jilin	1	<0.1%
Gansu	1	<0.1%
Hong Kong	1	<0.1%

Table S2. Model fit statistics for latent class analysis (LCA) models with different numbers of classes among MSM participants

Model	BIC	Entropy	Percent
2-class	8851.4	4.276	0.994
3-class	8835.6	4.242	0.996
4-class	8833.4	4.209	0.997
5-class	8865.4	4.191	0.997

BIC: Bayesian Information Criterion; CE: classification entropy. The optimal number of classes was selected based on the lowest BIC, highest CE, and class interpretability.

Table S3. Conditional probabilities of each behavioral indicator by latent class (*n* = 1,022).

**Behavioral Indicator**	**Response**	**Class 1 (Low-partner, high-risk, *n* =137)**	**Class 2 (Multi-partner, proactive, *n* =318)**	**Class 3 (Low-risk, conservative, *n* =567)**
**≥2 regular male partners**	Yes	0.01	0.68	0.1
	No	0.99	0.32	0.9
**≥2 casual male partners**	Yes	0	0.83	0.09
	No	1	0.17	0.91
**Condomless sex**	Yes	0.82	0.74	0.42
	No	0.18	0.26	0.58
**Group sex participation**	Yes	0.58	0.44	0.02
	No	0.42	0.56	0.98
**Frequent STD testing (≥1/3 months)**	Yes	0.19	0.7	0.43
	No	0.81	0.3	0.57
**PrEP LAI willingness**	Interested	0.59	0.83	0.64
	Uninterested	0.41	0.17	0.36
**PrEP awareness**	Aware	0.7	0.96	0.94
	Unaware	0.3	0.04	0.06
**Sexual orientation disclosure**	Yes	0.14	0.1	0.23
	No	0.86	0.9	0.77
